# Expression profiles of cholesterol metabolism-related genes are altered during development of experimental autoimmune encephalomyelitis in the rat spinal cord

**DOI:** 10.1038/s41598-017-02638-8

**Published:** 2017-06-02

**Authors:** Irena Lavrnja, Kosara Smiljanic, Danijela Savic, Aleksandra Mladenovic-Djordjevic, Katarina Tesovic, Selma Kanazir, Sanja Pekovic

**Affiliations:** Department of Neurobiology, Institute for Biological Research “Sinisa Stankovic” University of Belgrade, Belgrade, Serbia

## Abstract

Increased evidence suggests that dysregulation of cholesterol metabolism may be a key event contributing to progression of multiple sclerosis (MS). Using an experimental autoimmune encephalomyelitis (EAE) model of MS we revealed specific changes in the mRNA and protein expression of key molecules involved in the maintaining of cholesterol homeostasis in the rat spinal cord: 3-hydroxy-3-methylglutaryl-coenzyme-A reductase (HMGCR), apolipoprotein E (ApoE) and cholesterol 24-hydroxylase (CYP46A1) during the course of disease. The presence of myelin lipid debris was seen only at the peak of EAE in demyelination loci being efficiently removed during the recovery period. Since CYP46A1 is responsible for removal of cholesterol excess, we performed a detailed profiling of CYP46A1 expression and revealed regional and temporal specificities in its distribution. Double immunofluorescence staining demonstrated CYP46A1 localization with neurons, infiltrated macrophages, microglia and astrocytes in the areas of demyelination, suggesting that these cells play a role in cholesterol turnover in EAE. We propose that alterations in the regulation of cholesterol metabolism at the onset and peak of EAE may add to the progression of disease, while during the recovery period may have beneficial effects contributing to the regeneration of myelin sheath and restoration of neuronal function.

## Introduction

Multiple sclerosis (MS) is a progressive, neurodegenerative disease of the central nervous system (CNS) that generally affects young adults in the prime of their life. MS is thought to be evoked by autoreactive T cells that have role to guide phagocytes to degrade myelin sheath, leading to demyelination and axonal loss. In MS and its best characterized animal model, experimental autoimmune encephalomyelitis (EAE), T helper cells^1^ play the main role in initiation of disease, while macrophages are the major effector cells that dominate within inflammatory infiltrates^2^. Besides infiltrated macrophages, perivascular and meningeal macrophages, together with microglia induce demyelination and secrete pro-inflammatory mediators that contribute to vicious cycle of inflammation^3^. On the other hand, local macrophages/microglia are crucial players in limitation of the autoimmune response and/or reparation of the CNS tissue^3, 4^. In addition, microglia through recruitment of astrocytes is able to regulate myelin clearance^5^. Also, astrocytes can exacerbate inflammation with ensuing demyelination in some phases of disease or may promote migration of oligodendrocyte precursor cells (OPCs) and remyelination^6^.

Recently, it was proposed that MS is rather metabolic disorder than a disease of the immune system, that involves a dysfunction of lipid metabolism^7^, especially of cholesterol^8, 9^. The blood-brain barrier (BBB) renders homeostasis of CNS cholesterol independent of circulating cholesterol^10, 11^. The majority of CNS cholesterol is localized in myelin sheaths^10^, being an essential constituent of the myelin membrane^12^. Apart from being an important structural component of the CNS, cholesterol is also indispensable for myelination during CNS maturation, modulation of dendrite outgrowth and microtubule stability, as well as synaptogenesis^13^. Cholesterol homeostasis is a prerequisite for proper CNS functioning and its preservation is maintained through the sophisticated regulation of synthesis, transport, and elimination of excessive cholesterol from the brain. Cholesterol synthesis is accomplished through the action of endoplasmic reticulum-bound 3-hydroxy-3-methylglutaryl-coenzyme-A reductase (HMGCR), that is in control for the conversion of 3-hydroxy-3-methylglutarylcoenzyme-A into mevalonate^14^. Apolipoprotein E (ApoE) has a role in recycling and intercellular transport of cholesterol in the brain^15, 16^. Additionally, it has been demonstrated to take part in antigen presentation^17^ and maintenance of BBB integrity^18–20^, which is critical for the regulation of immune cell entry into the CNS. The major cholesterol elimination pathway is the conversion of cholesterol into 24(S)-hydroxycholesterol (24-OHC), which crosses the BBB, enters the circulation, and is eliminated by the liver^21, 22^. The enzyme responsible for this conversion is the cholesterol 24S-hydroxylase (CYP46A1), a member of microsomal cytochrome P450 family.

Expression of CYP46A1 has been documented in multiple regions of the brain. It is a neuron-specific enzyme, predominantly expressed in neuronal cell bodies and dendrites of only a subset of neurons, suggesting that constant cholesterol synthesis and turnover are crucial for the specific functions of these selected cells^23^. Hitherto, there are only few articles showing atypical CYP46A1 expression, generally associated with assorted pathological conditions. Bogdanovic and colleagues^24^ detected CYP46A1 positive immunostaining in glial cells in brain samples of patients with an advanced Alzheimer’s disease. Similarly, CYP46A1 positive astrocytes, surrounding the lesion site were detected after the hippocampal kainate injury^25^. In our previous study^26^, we have reported that traumatic brain injury induces the long-lasting upregulation of CYP46A1 protein expression at the lesion site, which was attributed to microglia and astrocytes in a time-dependent manner.

Active MS lesions are characterized by extensive demyelination and contain large numbers of macrophages filled with myelin. Myelin taken up by macrophages is degraded generating oxysterols, which could have a role in promoting autoimmunity as signal molecules^27^. Additionally, cholesterol may promote inflammatory mediators production by macrophages^28^, generating vicious cycle of inflammation. Demyelination occurring as a consequence of inflammation and neurodegeneration may transiently increase levels of 24S-hydroxycholesterol in the circulation during acute stage of MS^29^. However, chronic neurodegeneration leads to lowered levels of oxysterols, cholesterol precursors and apolipoprotein E in the circulation^8, 9, 30^. Therefore, the metabolism of myelin lipids, such as cholesterol, is indicated as a potential therapeutic target^28^.

Although a perturbed cholesterol metabolism plays an important role in the pathology of MS, little is known about the specific changes in the expression of key regulators of cholesterol homeostasis. Therefore, using a rat EAE model, we analyzed the mRNA and protein expression pattern of HMGCR, ApoE and CYP46A1 in the spinal cord during the onset, progression and resolution of disease. Additionally, a detailed cellular profiling of CYP46A1 expression during EAE is presented. Our results revealed specificity of changes in the expression of cholesterol-related genes (HMGCR, ApoE, CYP46A1) during the course of the disease. In addition, we have found regional and temporal shift in distribution of CYP46A1 in the spinal cord during EAE showing its presence not only in neurons, but also in infiltrated macrophages, microglia and astrocytes.

## Results

### Clinical Symptoms of EAE

**Figure 1:**
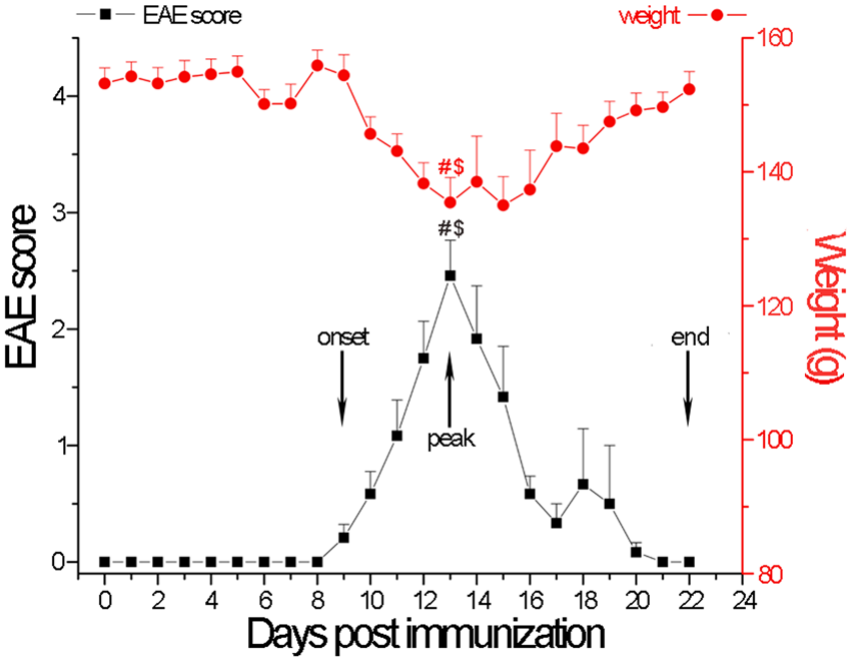
Disease severity scores. EAE was induced in female Dark Agouti rats following immunization with rat spinal cord homogenate (50% w/v emulsion in saline) emulsified in complete Freund’s adjuvant. Naïve rats served as a control. Animals were scored for neurological signs of EAE according to the standard 0–5 EAE grading scale (black line): 0 - unaffected; 1 - tail atony; 2 - hind limb weakness; 3 - complete hind limb paralysis; 4 - tetraplegic; 5 - moribund state or death. Progress of EAE was associated with body weight loss, with maximal loss that coincided with paralysis at the peak of disease (red line). Animals from EAE group (10/group) were sacrificed at the onset (D9), peak (D13) and end (D22) of disease. Data are expressed as the mean clinical score ± SEM (10 rats per group). Differences between the experimental groups were tested using ANOVA followed by Bonferroni test. ^#^Peak vs. onset of EAE, ^$^Peak vs. end of EAEs (*P* < 0.05).

The animals developed acute monophasic disease with 100% incidence, following the sensitization by active immunization with whole spinal cord homogenate (Fig. 1). First signs of the disease appeared at 9 dpi (D9) and were designated as the onset of disease and reached a peak at D13. Clinical signs of EAE decreased afterwards during the period of recovery, and all rats completely recovered at D22 (end of disease). Development of EAE signs was accompanied by body weight loss with maximal loss that coincided with paralysis at the peak of disease (Fig. 1).

### The progression of EAE alters expression profiles of HMGCR, АpoЕ and CYP46A1 in the spinal cord

**Figure 2:**
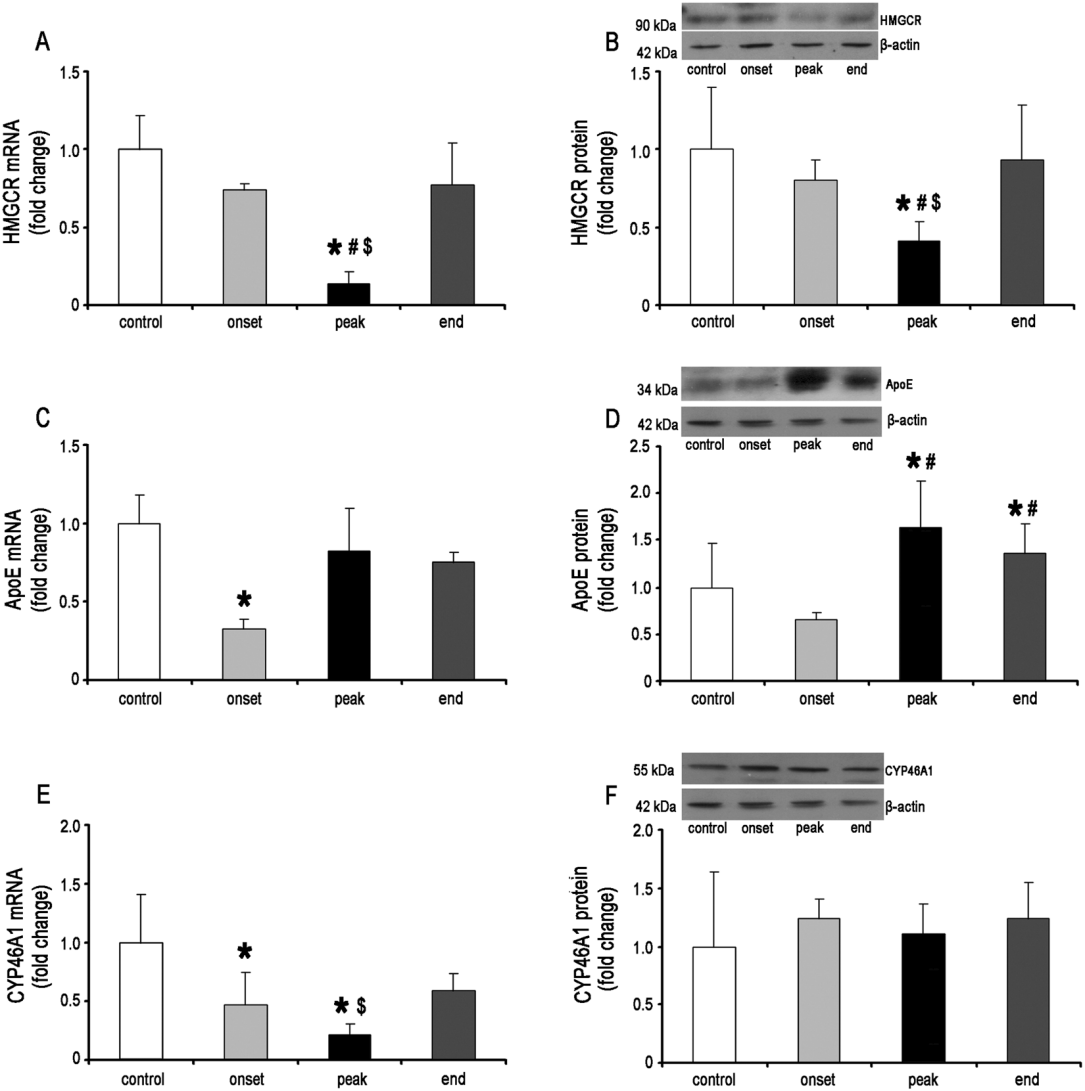
HMGCR, ApoE and Cyp46A1 expression during EAE in the rat spinal cords. The levels of (**A**) HMGCR, (**C**) ApoE and (**E**) CYP46A1 mRNAs isolated from lumbar part of spinal cords of control and EAE rats at the onset, peak and the end of disease were assessed by Real-time PCR. β-actin was used as an endogenous control. The protein levels of (**B**) HMGCR, (**D**) ApoE and (**F**) CYP46A1 were measured by Western blots. Shown are representative blots for each protein with β-actin used as a loading control. Both mRNA and protein levels are expressed as a fold change relative to the values in spinal cords of control animals. The data represent the mean ± SD.*P < 0.05 vs. control; ^#^ *P* < 0.05 vs. onset; ^$^ *P* < 0.05 vs. end of EAE.

Accumulating evidence underscores the capability of perturbed cholesterol homeostasis to modulate inflammation and potentially promotes autoimmune disease^27, 28^. Therefore, we next analyzed the expression pattern of key molecules involved in biosynthesis (HMGCR), recycling (ApoE), and degradation (CYP46A1) of cholesterol in the course of EAE using real-time PCR and Western blot analysis (Fig. 2).

At the onset of disease there were no significant changes in HMGCR mRNA level compared to control. Interestingly, although the levels of HMGCR mRNA was significantly reduced (~7 fold) at the peak of disease, it was returned to control level at the end of disease (Fig. 2A). Correspondingly, the prominent decrease of the HMGCR at the protein level was only detected at the peak of EAE (Fig. 2B).

ApoE mRNA levels was significantly decreased at the onset, while at the peak and the end of EAE it returned to control levels (Fig. 2C). In contrast, ApoE protein level exerts the most pronounced changes at the peak of disease. ApoE protein expression was significantly increased compared to the levels in controls and at the onset of disease and remained elevated throughout the recovery period until the end of disease (Fig. 2D).

CYP46A1 expression profile revealed a significant decrease in mRNA levels at all investigated time points, being at the lowest level at the peak of disease (Fig. 2E). Particularly, when compared to control, CYP46A1 mRNA levels decreased 2-, 5- and 2-fold at the onset, peak and the end of EAE, respectively. In contrast, CYP46A1 protein levels remained unchanged during the course of disease (Fig. 2F).

### CYP46A1 expression localizes in the areas of demyelination during EAE

**Figure 3:**
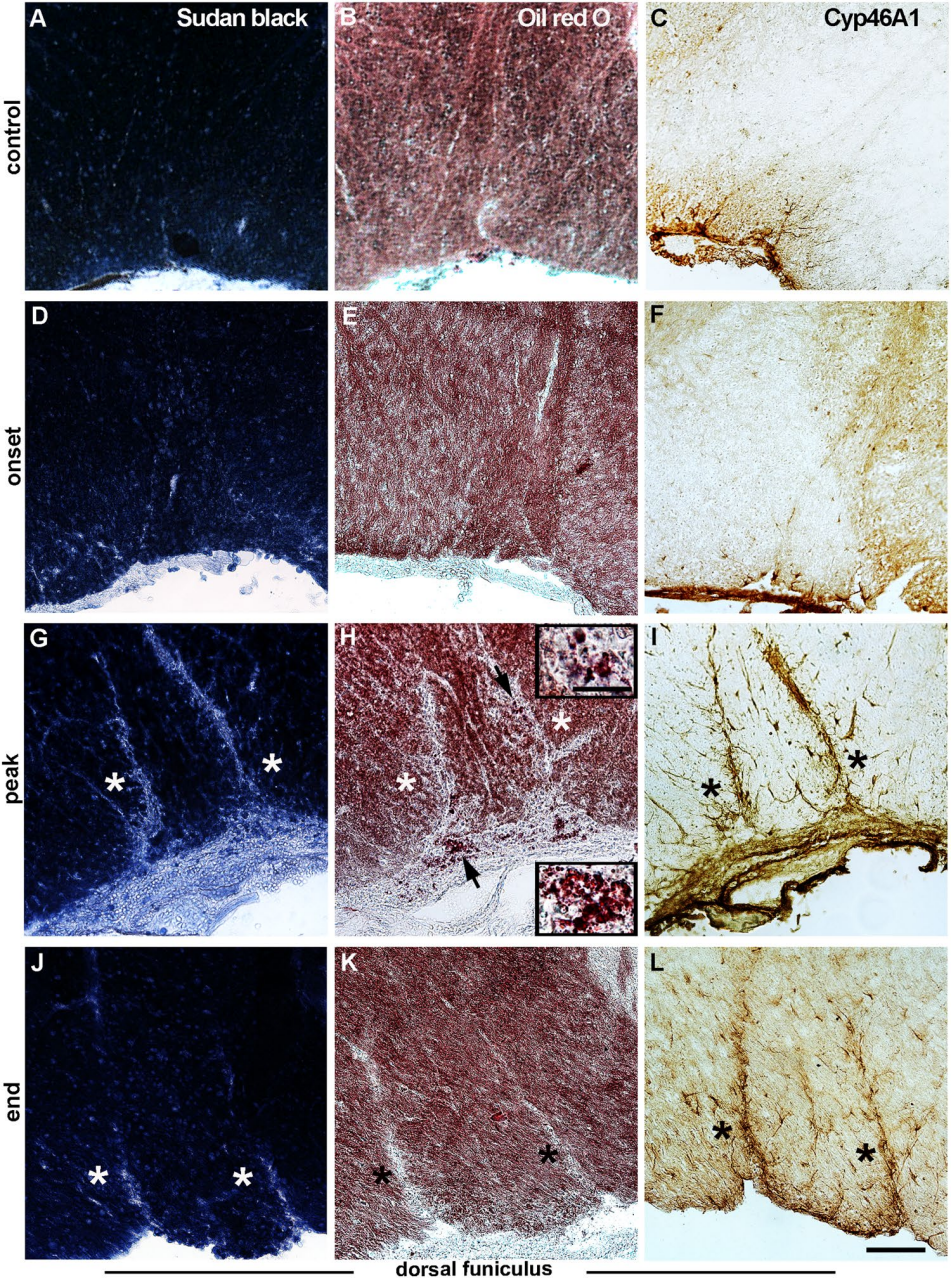
Cyp46A1 expression within the areas of demyelination and lipid degradation in the spinal cords of EAE animals. 20 µm thick transverse sections of the lumbar part of spinal cord were used. (**A,D,G,J**) Demyelination processes during the course of disease were visualized with Sudan black B. (**A**) In the control sections and (**D**) at the onset of EAE myelin was compact and stained black. (**G**, asterisks) Pale blue stained demyelination loci were detected at the peak and (**J**, asterisks) at the end of disease in dorsal funiculus. (**B,E,H,K**) The presence of lipid debris during the development of EAE was detected with Oil-Red-O. (**H**, asterisks, insets) Myelin lipid debris coincided with the areas of demyelination and was seen only at the peak of EAE. (**K**, asterisks) At the end of EAE the lipid debris were removed. (**C,F,I,L**) CYP46A1 immunostaining in the dorsal funiculus of rat spinal cord was performed using rabbit anti-Cyp46A1 antibody T623 and was visualized by DAB-HRP staining. (**C**) In the control sections and (**F**) at the onset of EAE a few CYP46A1^+^ cells were scattered throughout the white matter. (**I**, asterisks) The most pronounced CYP46A1 staining was detected at the peak of disease in the areas of demyelination and lipid degradation. (**L**, asterisks) At the end of EAE decreased CYP46A1 expression was still associated with the plaques of demyelination. Scale bar = 100 μm for the large panels and 25 μm for the magnified insets.

To visualize demyelination process during the course of disease, spinal cords from EAE animals were stained with Sudan black and compared with controls. At the onset of EAE (Fig. 3D), as well as in the control sections (Fig. 3A) myelin was compact and stained black. At the subsequent time points, pale blue staining of demyelination loci was observed in the dorsal funiculus, preferentially in the gracile fasciculus (Fig. 3G,J).

In order to detect the presence of lipid debris during the development of EAE consecutive sections were stained with Oil-Red-O (Fig. 3B,E,H,K). Myelin lipid debris was seen only at the peak of EAE (Fig. 3H and insets) and coincided with the areas of increased myelin pallor (Fig. 3G). The absence of lipid laden myelin debris at the end of EAE (Fig. 3K) suggests their efficient clearance during the recovery period.

Given the important role of CYP46A1 in the clearance of cholesterol, the main lipid constituent of the membranes, we next performed histological examination of CYP46A1 staining (Fig. 3C,F,I,L) and revealed its presence in the areas of demyelination and lipid degradation. The most prominent CYP46A1 expression was detected at the peak of disease and was associated with the plaques of demyelination in the gracile fasciculus (Fig. 3I). Interestingly, further inspection of CYP46A1 immunoexpression revealed its localization with different types of cells in the spinal cord sections of all experimental groups (Supplementary Fig. 2). In the white matter (dorsal, lateral and ventral) CYP46A1 immunoreactivity was mostly associated with the glia-like cells (Fig. 3C,F,I,L; Supplementary Fig. 2A–H), while in the gray matter it was predominantly present in neuronal cell bodies (Supplementary Fig. 2I–L). Next, to confirm CYP46A1 co-localization with particular type of cells and to reveal whether it is altered during the course of disease, we performed double immunofluorescence staining with specific cell type markers.

### CYP46A1 expression localizes in macrophages/microglia infiltration during the progression of EAE

**Figure 4:**
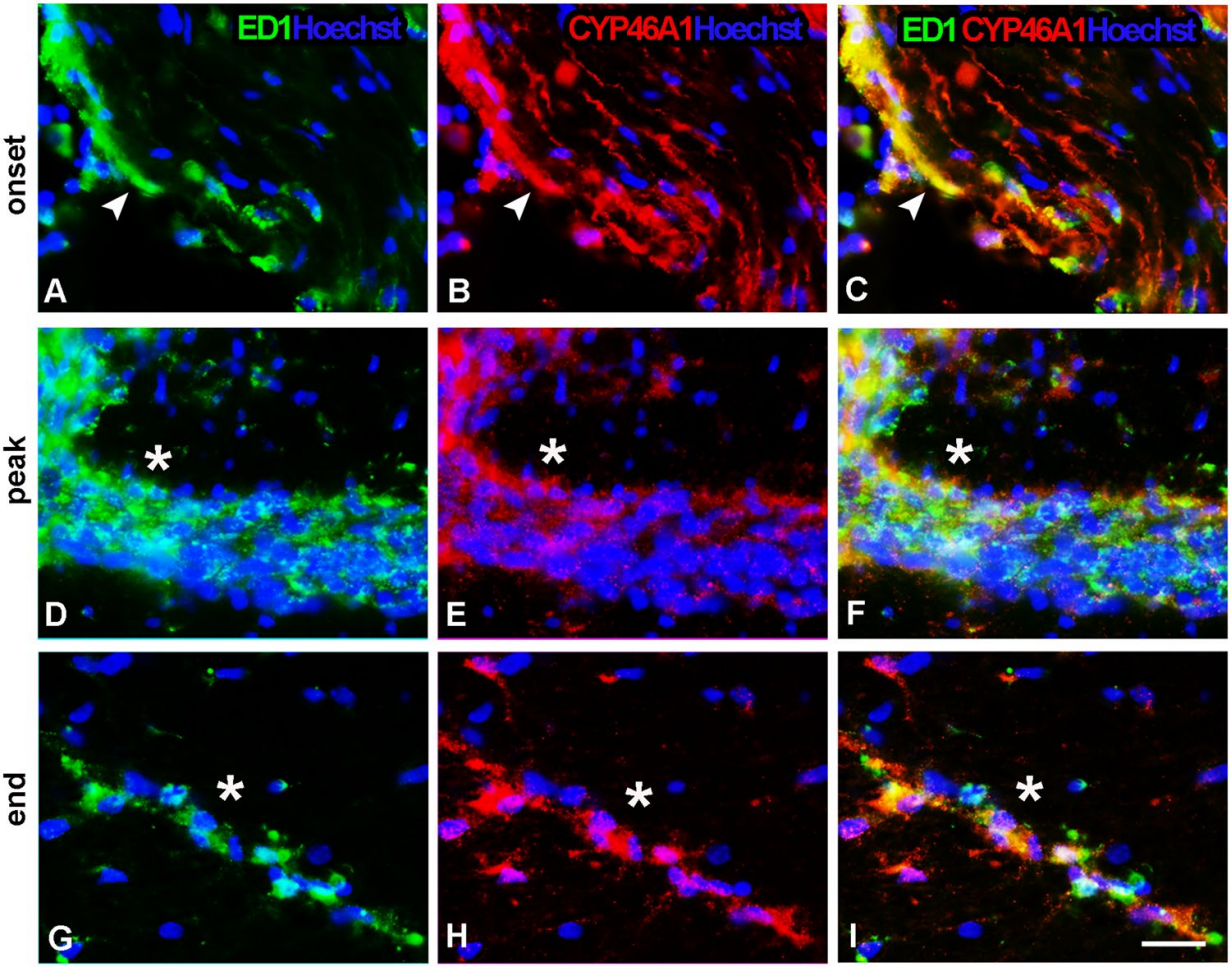
Infiltrating macrophage/microglia express CYP46A1 during the development of EAE. Cyp46A1 expression (red) in ED1-positive (green) macrophages and reactive microglia in the dorsal funiculus of spinal cord. Nuclei were visualized with Hoechst (blue) staining. (**A–C**, arrow head) At the onset of disease ED1-immunopositive cells were scattered along the pial surface and in perivascular space completely overlapped with CYP46A1-positive structures. (**D**, asterisk) During the peak of disease, clusters of round ED1^+^ cells accumulate within the area of demyelination. (**E,F**, asterisk) Strong CYP46A1 staining could be obsereved with ED1-positive infiltrates. (**G–I**, asterisk) At the end of the disease, number of ED1^+^ cells was reduced and they were dispersed among CYP46A1^+^ structures. Scale bar = 20 µm.

CYP46A1 presence in the macrophages/microglia over the course of EAE was demonstrated by CYP46A1/ED1 double immunofluorescence, using ED1 as a marker of macrophages and reactive microglia. In the control spinal cord sections no CYP46A1/ED1 immunoreactivity was detected (data not shown). At the onset of disease, ED-1 immunopositive cells were primarily observed in a close proximity to the pial surface of the spinal cord and in perivascular space scattered between CYP46A1 positive structures predominantly in dorsal funiculus of spinal cord (Fig. 4A–C). In ventral and lateral regions of the spinal cords (Supplementary Fig. 3A–F), CYP46A1 expression in ED1^+^ macrophages was detected exclusively in the areas of infiltration. During the peak of disease, numerous amoeboid cells were ED1^+^. Clusters of round ED1^+^ cells were observed throughout the spinal cord parenchyma, especially at gracile fasciculus in the area of demyelination (Fig. 4D). Strong CYP46A1 staining (Fig. 4E) has been shown to localize in ED1^+^ macrophages within the areas of macrophage infiltration in spinal cord tissue (Fig. 4F). Similarly, in the ventral and lateral regions of spinal cords, CYP46A1/ED1 immunoreactivity overlaps with macrophage infiltrates (Supplementary Fig. 3G–L). At the end of disease, sparse ED1^+^ cells were dispersed among CYP46A1 positive structures in dorsal funiculus (Fig. 4G–I). Comparable pattern of expression was observed in ventral and lateral regions of the spinal cords (Supplementary Fig. 3M–R).

### CYP46A1 is expressed by resident microglia at the peak and the end of EAE

**Figure 5:**
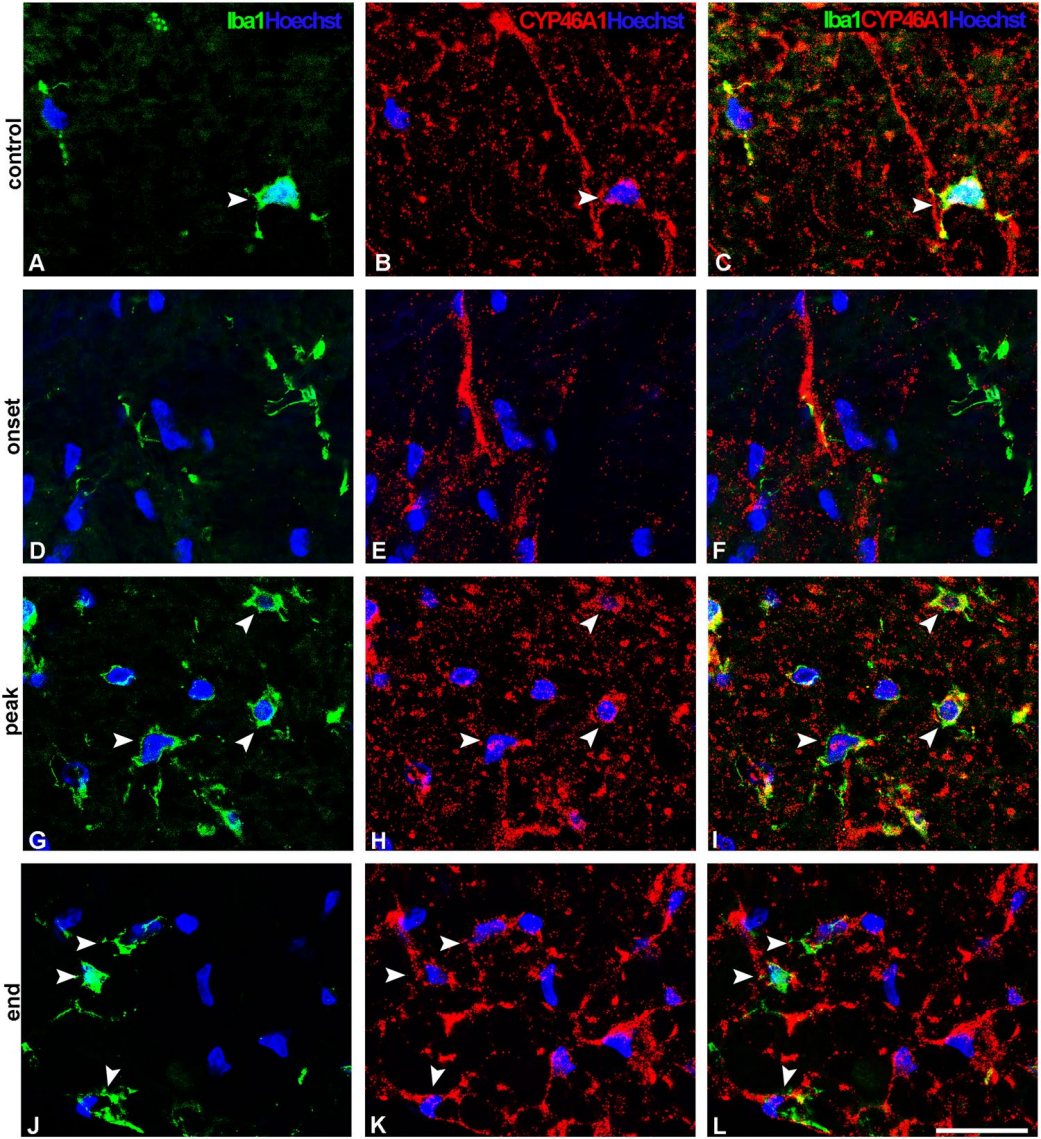
CYP46A1 expression in Iba1^+^ resident microglia correlates with clinical signs of EAE. Double immunofluorescence was used to reveal the presence of CYP46A1 (red) in the resident microglia stained with Iba1 (green) in the dorsal funiculus of the spinal cord. Cell nuclei were visualized with Hoechst (blue) staining. (**A–C**, arrow head) In the control sections, Iba1/CYP46A1-positive ramified microglia were sporadically present in the dorsal funiculus. (**D–F**) Absence of CYP46A1 staining in Iba1^+^ microglia at the onset of disease. (**G–I**, arrow heads) At the peak of disease CYP46A1 immunoreactivity was observed in numerous hypertrophied Iba1^+^ cells. (**J–L**, arrow heads) At the end of the disease only a few Iba1^+^ cells, with more resting-like morphology, express CYP46A1. Scale bar = 20 µm.

To reveal the presence of CYP46A1 in the resident microglia the spinal cord sections were stained with Iba1, which is expressed both in resting and reactive microglia. In the control sections, ramified Iba1 and CYP46A1 positive cells were sporadically present in dorsal and ventral funiculus (Fig. 5A–C; Supplementary Fig. 4A–C) as well as in lateral regions of lumbosacral spinal cord (Supplementary Fig. 4D–F). At the onset of disease, no CYP46A1/Iba1 positive microglial cells were detected in dorsal funiculus (Fig. 5D–F), while in ventral and lateral spinal cord regions faint CYP46A1 immunoreactivity was sporadically present in microglial cells (Supplementary Fig. 4G–L). The peak of the disease was characterized by CYP46A1 immunoreactivity localized in numerous hypertrophied Iba1^+^ cells in dorsal funiculus (Fig. 5G–I), ventral and lateral regions of spinal cord (Supplementary Fig. 4M–R). Iba1^+^ cells changed their morphology to resting form in a white matter of spinal cord at the end of the disease (Fig. 5J–L, Supplementary Fig. 4S–X), and only a few Iba1^+^ cells expressed CYP46A1.

### Reactive astrocytes express CYP46A1 during the course of EAE

**Figure 6:**
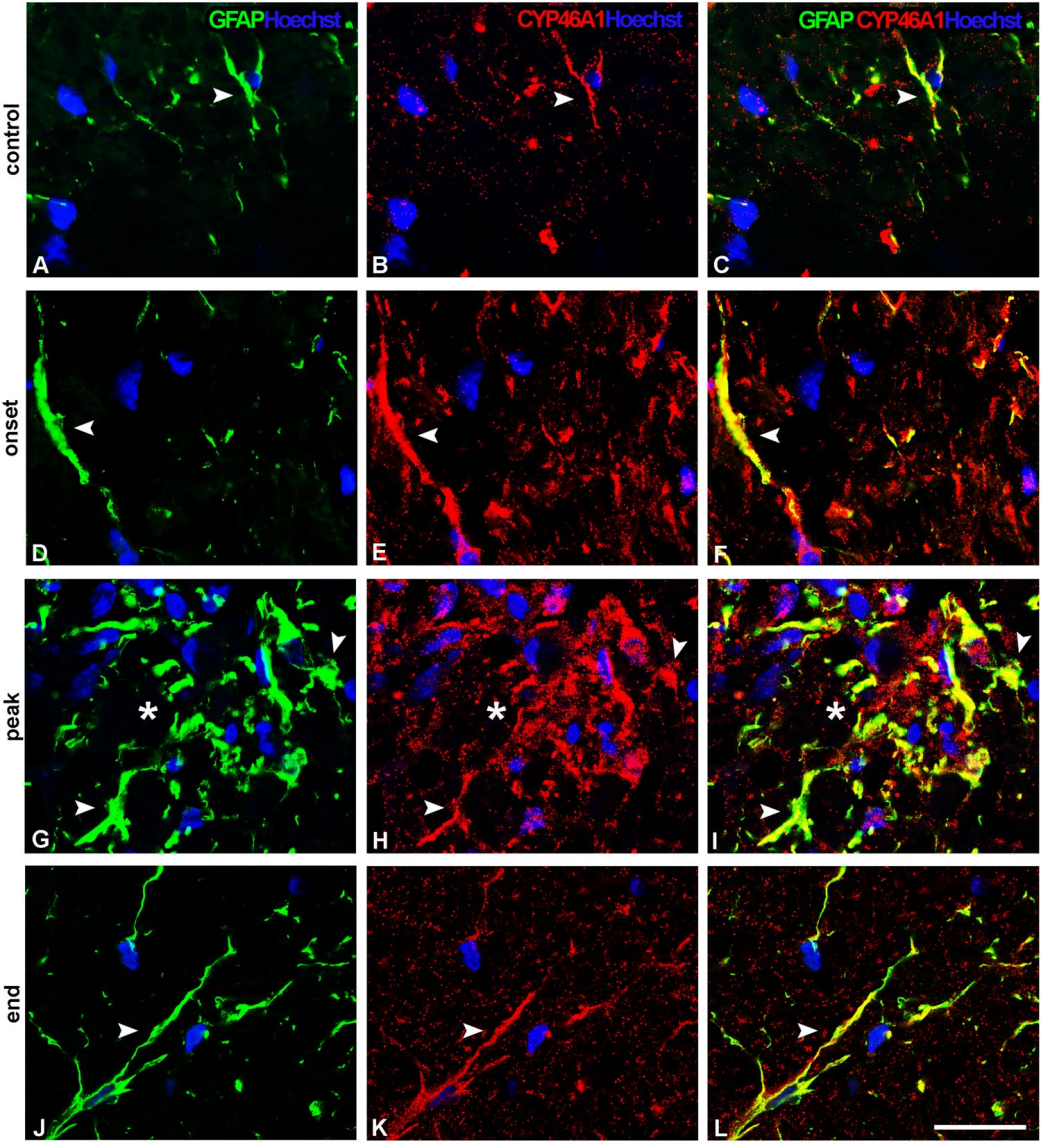
CYP46A1 localize with reactive astrocytes in the course of EAE within the white matter of the lumbar spinal cord. GFAP^+^ astrocytes (green) express Cyp46A1 (red) in the dorsal funiculus of the spinal cord. Nuclei were visualized with Hoechst (blue) staining. (**A–C**, arrow head) Fibrous astrocytes in the control sections display CYP46A1 immunoreactivity. (**D–F**, arrow head) At the onset of disease, astrocytes with elongated extensions express CYP46A1. At the peak of EAE astrocytes in the close vicinity to the area of demyelination undergo the most prominent phenotypic changes. (**G**, arrow head) Hypertrophied astrocytes with enlarged intertwining processes formed the scar border around the areas of demyelination (asterisk), and (**H,I**, arrow head, asterisk) display complete overlapping with strong CYP46A1 immunostaining. (**D–F**, arrow head) Hypertrophied astrocytes with elongated processes expressing CYP46A1 were observed at the end of EAE as well. Scale bar = 20 µm.

**Figure 7:**
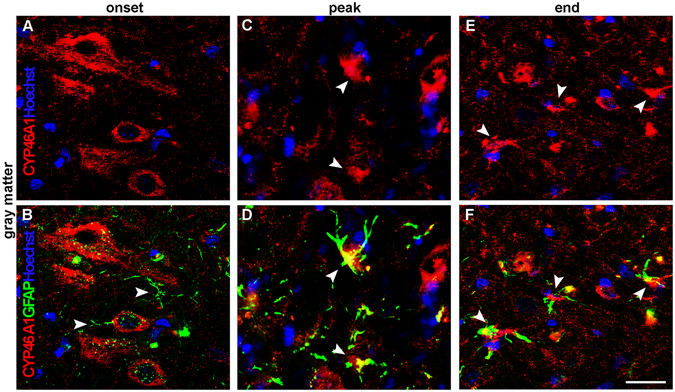
Localization of CYP46A1 in reactive astrocytes within the gray matter of the lumbar spinal cord during the course of EAE. Nuclei of cells were visualized with Hoechst (blue) staining. (**B**, arrow head) At the onset of disease, no overlapping of CYP46A1 (red) with GFAP^+^ (green) astrocytes was seen. (**C,D**, arrow heads) In contrast, at the peak of EAE, intensive CYP46A1 staining was observed in GFAP^+^ reactive astrocytes. (**E,F**, arrow heads) The same immunostaining pattern of CYP46A1 expression in the reactive astrocytes with large cell bodies and short extensions was demonstrated at the end of EAE. Scale bar = 20 µm.

Control sections display CYP46A1 expression in fibrous astrocytes sporadically present throughout the white matter, including dorsal funiculus, ventral and lateral regions of spinal cord (Fig. 6A–C; Supplementary Fig. 5A–F), while in the gray matter no CYP46A1/GFAP overlapping was seen (data not shown). At the onset of disease, astrocytes with elongated extensions expressing CYP46A1 were present in dorsal (Fig. 6D–F), ventral and lateral regions of the spinal cord (Supplementary Fig. 5G–L). However, CYP46A1staining in GFAP^+^ astrocytes in the gray matter could not be observed (Fig. 7B). The most prominent phenotypic changes of astrocytes occurred at the peak of disease adjacent to the area of demyelination. Hypertrophied astrocytes with enlarged intertwining processes formed the scar border (Fig. 6G, Supplementary Fig. 5M,P). Densely populated GFAP^+^ astrocytes expressing CYP46A1 were seen in dorsal funiculus (Fig. 6I). Similarly, CYP46A1^+^/GFAP^+^ hypertrophied astrocytes with short processes comprised ventral and lateral regions of the spinal cord (Supplementary Fig. 5O,R). In the gray matter, intensive CYP46A1 staining was present in GFAP^+^ reactive astrocytes (Fig. 7C,D). At the end of EAE, hypertrophied astrocytes with elongated processes expressing CYP46A1 were observed in the gracile fasciculus (Fig. 6J–L). Similarly, in the ventral and lateral regions of the spinal cord, astrocytes display overlapping signal with CYP46A1 (Supplementary Fig. 5U –X). In the gray matter, strong CYP46A1 staining was localized in reactive astrocytes with large cell bodies and short extensions (Fig. 7E,F).

### Decreased neuronal expression of CYP46A1 coincides with the peak of EAE

**Figure 8:**
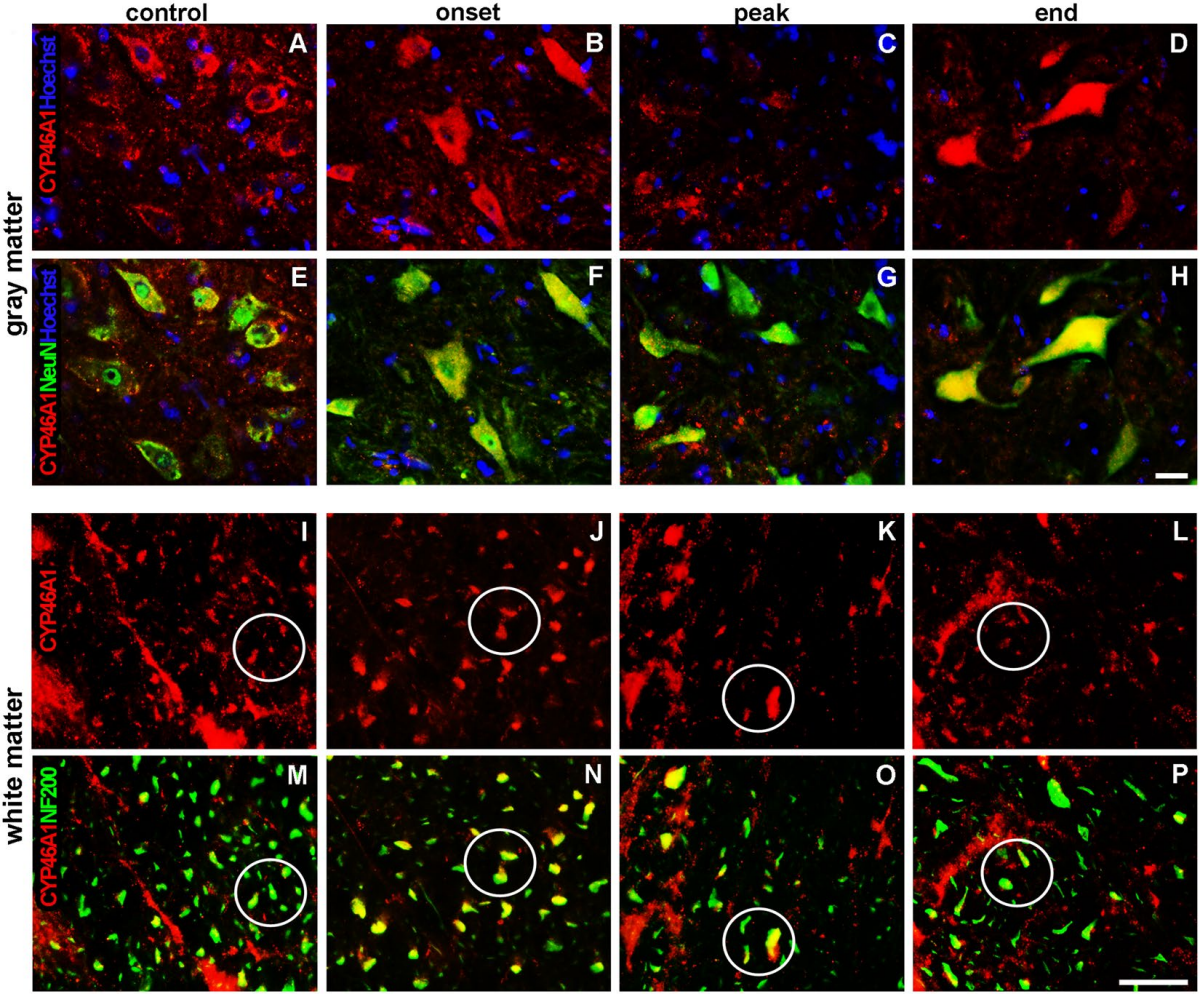
Neuronal expression of CYP46A1 was decreased at the peak of EAE. (**A–H**) Nuclei of all cells were visualized with Hoechst (blue) staining. Double immunolabeling of spinal cord sections with NeuN (green), a marker of neuronal nuclei, revealed obvious overlapping with CYP46A1 (red). (**A,E,B,F,D,H**). In the gray matter of all examined groups strong CYP46A1 immunoreactivity was observed in the neuronal somata, except (**C,G**) at the peak of EAE when intensity of CYP46A1 staining was reduced. (**M–P**) In the white matter, double immunofluorescence with anti-NF200 antibody (green) revealed the presence of CYP46A1 (red) immunoreactivity in the axons during the course of EAE and confirmed that (**K,O**) at the peak and (**L,P**) at the end of disease CYP46A1 staining is decreased due to axonal loss. Scale bar = 20 µm.

Double immunolabeling of spinal cord sections with NeuN, as a marker of neuronal nuclei, revealed a clear staining for CYP46A1 (Fig. 8E–H). Prominent CYP46A1 immunoreactivity in neuronal somata was observed in the gray matter of all examined groups (Fig. 8A,B,D), except at the peak of disease when intensity of CYP46A1 staining was lowered (Fig. 8C).

In the white matter, double immunofluorescence with anti-NF200 antibody revealed the presence of CYP46A1 immunoreactivity in the axons during the course of disease (Fig. 8M–P) and confirmed that decrease in CYP46A1 staining at the peak and, to a lesser extent, at the end of disease (Fig. 8K,L) is due to axonal loss (Fig. 8O,P).

## Discussion

In the present study, using experimental autoimmune encephalomyelitis (EAE) as an animal model for MS, we found a link between the expression pattern of key molecules involved in the biosynthesis (HMGCR), recycling (ApoE) and degradation (CYP46A1) of cholesterol and the progression and resolution of EAE in the rat spinal cord. This implies that maintaining a balance between cholesterol utilization and excretion is very important during EAE. In addition, we have shown that aside from its expression in neurons, CYP46A1 was localized within different types of glial cells in a time-dependent manner.

Numerous studies over the past decades have reported associations between MS disease outcome and levels of cholesterol precursors, oxysterols and ApoE in circulation and CSF^8, 9, 30^, indicating the need for closer insight into the cholesterol homeostasis in MS pathogenesis. However, to the best of our knowledge, the cholesterol homeostasis in the spinal cords during EAE has been poorly addressed so far.

Given that HMGCR is a rate limiting enzyme in the cholesterol biosynthesis in this study we examined the relationship between its gene and protein expression and the clinical scores of EAE. We have observed that the most prominent down-regulation of HMGCR gene and protein expression occurred in the spinal cord at the peak of disease, when inflammation and demyelination are the most pronounced. Similarly, reduced HMGCR gene expression was detected at the peak of MOG-induced EAE in female DA rats^31^ as well as in lysolecithin model of demyelination^32^. Recently, it was shown that products of the cholesterol biosynthesis pathways are interrelated with myelin genes expression^33^. Pertinent to the later, the observed suppression of HMGCR expression may be correlated with persistent down-regulation of transcripts for several myelin proteins like MOG, MBP, MAG, during EAE^31^. Furthermore, alteration in HMGCR expression is associated with misdirected OPC migration, leading to hypomyelination^33^. Since HMGCR expression is negatively regulated by cholesterol content, the lower expression of both HMGCR transcript and protein detected at the peak of EAE may be the consequence of high cholesterol concentration due to demyelination in spinal cords of EAE animals, reducing the need for cholesterol biosynthesis. In contrast, at the end of disease, the observed increase in HMGCR expression is probably due to the increased demand for cholesterol needed for re-myelination during the recovery period.

ApoE plays a central role in lipid transport and cholesterol metabolism in the CNS. It exerts pleiotropic effects during EAE, both protective^34, 35^ and harmful^36, 37^. Here, we have observed the significant down-regulation of ApoE expression at the onset of disease, followed by elevated levels at the peak and the end of disease. It is plausible to presume that elevation of ApoE levels at the peak and the end of disease may be due to the increased number of glial cells that we have detected in the spinal cord of EAE animals, particularly in and around the areas of demyelination. Our results are in agreement with Shin *et al*.^36^, who also observed elevated expression of ApoE during EAE and suggested that invading cells may be responsible for part or all of the enhanced ApoE. In line with this, in active MS lesion, the increased ApoE expression was found in astrocytes and macrophages^38^, supporting the ApoE involvement in lipid trafficking needed for neuronal repair^39, 40^. Indeed, it was shown that cholesterol released as a result of demyelination associates with ApoE-containing lipid particles and is available for reutilization^41^. In addition, ApoE acts to remove cholesterol from damaged neurons in order to restore axonal properties, with concomitant reduction of inflammation^9^. Furthermore, Apo E deficiency leads to massive infiltration within CNS during EAE^35^ and increased permeability of BBB^42^, indicating that ApoE has strong anti-inflammatory effect on EAE progression^34^. Indeed, elevated expression of ApoE that we have observed at the end of EAE may be direct response to demyelination, as it is proposed by Schrewe *et al*.^43^.

CYP46A1 is the major regulator of CNS cholesterol elimination^44^. So far, no studies have been carried out to evaluate possible correlations between the CYP46A1 mRNA and protein content in the rat spinal cord and onset, progression and resolution of EAE. In this study, we have observed reduction of CYP46A1 mRNA levels at the onset of disease, which was followed by further decrease at the peak of EAE. However, this drop in the CYP46A1 mRNA expression was returned to the control level during the period of recovery. Notably, protein content of CYP46A1 remains unchanged during the course of the disease. Given that CYP46A1 is predominantly produced by neurons, the observed decrease in CYP46A1 mRNA possibly reflects the loss of neuronal cells which is the most pronounced at the peak of EAE. On the other hand, unchanged protein content points that other type of cells, presumably glial cells, contribute to CYP46A1 production. Indeed, we have shown the presence of CYP46A1 in infiltrated macrophages, microglia and astrocytes in the areas of prominent demyelination in the course of EAE. Similarly, it was shown that decrease of CYP46A1 in neuronal cells is at least in part compensated by an induction of the enzyme in glial cells^24^. In addition, Teunissen *et al*.^45^ proposed that infiltrating macrophages participated in CYP46A1 production during EAE. During EAE, activated microglia and infiltrated macrophages are involved in the clearance of myelin debris and apoptotic cells from the CNS. Moreover, myelin debris removal is prerequisite for remyelination process. In our study, the appearance of lipids and lipoproteins as a part of destroyed myelin was detected at the peak of disease as confirmed by Oil Red-O staining. The absence of lipid components at the end of disease implies that the remains of degraded myelin were efficiently removed by activated microglia. Interestingly, we have demonstrated heightened CYP46A1 expression within the areas of demyelination during EAE. Furthermore, aside from neurons CYP46A1 immunoreactivity can be observed in infiltrating and resident macrophages/microglia and astrocytes.

Previously, we and others have shown that activated macrophages/microglia express CYP46A1 in traumatic brain injury^26, 46^. Additionally, it was suggested that infiltrating macrophages express CYP46A1 during EAE^45^. Although it is difficult to distinguish activated resident microglia from infiltrating macrophages, a strong correlation between monocyte infiltration and progression to the paralytic stage of EAE suggests that the infiltrating macrophages are crucial for the pathogenesis^47^. Activated microglia can occur at early stage of tissue injury and usually precedes the formation of demyelinated plaques, which contribute to progressive neurodegeneration^48^. Our study confirmed the presence of CYP46A1 in infiltrated macrophages during development of EAE and further demonstrated its region-specific pattern of expression in spinal cords. The strongest CYP46A1 staining in activated macrophages/microglia was found at the peak of disease in the areas of demyelination. During recovery process, the population of infiltrating monocytes vanishes, while activated microglia enters the cell cycle and returns to quiescence^2^. Indeed, in our study microglia acquire resting form in recovery phase, with some cells still expressing CYP46A1.

The role of astrocytes in MS pathogenesis depends on various factors, including: the stage of the disease, the environment surrounding the lesion, and the interactions with other cell types, which may affect their state of activation^6^. During MS or EAE, astrocytes undergo proliferation and extensive hypertrophy of cell bodies, accompanied by thickening and elongation of their processes^49^. Activated astrocytes in EAE and MS are associated with the glial scar formation, where a physical border composed of hypertrophic astrocytes separates an area of CNS damage from normal appearing tissue^50^, resulting in the extracellular matrix composition changes, which prevent remyelination^51^. It was proposed that the availability of cholesterol is crucial for myelin membrane growth^52^ and astrocytes have been described as an important source of cholesterol for neurons^53^. Previously, we have shown an increase of GFAP gene and protein expression at the end of EAE, suggesting astrocytes involvement in neuroprotection^50^ and possibly in lipid trafficking^54^. The novel finding of this study is the association of CYP46A1 with reactive astrocytes surrounding demyelination plaques in the spinal cord of EAE rats, where those astrocytes are probably involved in clearance of axonal and myelin debris. Namely, immunofluorescence analysis revealed that radially-oriented GFAP^+^ astrocytes expressed CYP46A1 throughout the white matter of spinal cords during EAE. In the grey matter, besides obvious CYP46A1 expression in neuronal cell bodies, the abundant presence of this enzyme can be noticed in hypertrophic astrocytes at the peak and the end of disease. It is supposed that activated astrocytes in recovery phase of EAE have a putative role in reducing inflammation and in protection of the neurons from oxidative stress^55^. Thus, the apparent localization of CYP46A1 in astrocytes at the end of disease, either in white or gray matter, possibly correlates with recovery process and remyelination. Those processes have high demand for cholesterol synthesis and CYP46A1 is needed in order to remove excess of cholesterol that fails to incorporate into the myelin membranes.

CYP46A1 expression was found in many types of neurons, where a majority of CYP46A1 expression was exclusively associated to neuronal somata, with lower levels found in dendrites^23, 26, 44, 56^. This study also revealed a strong CYP46A1 immunoreactivity in the neuronal somata in the grey matter at the onset and the end of EAE, which was lowered at the peak of disease probably due to neuronal degeneration. For the first time we have been shown the presence of CYP46A1 in axons. It is important to note pronounced axonal CYP46A1 immunoreactivity at the onset and the end of EAE and diminished immunoreactivity at the peak of the disease. This indicates that changes in pattern of CYP46A1 expression may reflect the degree of axonal damage. Up to now, positive CYP46A1 immunostaining of axons in the white matter was only seen in the normal human brain and in the brains of Alzheimer’s disease patients^24^. The authors proposed that CYP46A1 may be transported from the neuronal cell bodies to the periphery, suggesting that such mechanism may be of importance for maintaining the plasticity of the synapses. This assumption was recently confirmed by Moutinho *et al*.^13^, who report that CYP46A1 promotes neuronal outgrowth and increases synaptic markers.

Summarizing, the observed data indicate that alterations in the regulation of cholesterol metabolism during the onset and peak of EAE may add to the progression of disease, while during the recovery period may have the beneficial effects contributing to the regeneration of myelin sheaths and restoration of neuronal function. It appeared that gene and protein expression of key regulators of cholesterol homeostasis HMGCR, ApoE and CYP46A1 correlates with the clinical scores of disease. Cellular profiling of CYP46A1 expression in the spinal cord during EAE revealed that its expression is not exclusively in neuronal cells. The presence of CYP46A1 was confirmed in infiltrated macrophages, microglia and astrocytes, indicating that these cells in neuroinflammatory environment become sensitive to excess of cholesterol and play a role in cholesterol turnover. The fact that cholesterol is a crucial, rate-limiting factor for myelin growth and that its metabolites may have a role in promoting autoimmunity as inflammatory mediators, warrants further investigation of cholesterol metabolism.

## Materials and Methods

### Experimental animals, EAE induction and disease severity assessment

All experiments were conducted using two-month-old female rats of Dark Agouti inbred strain from the local colony. Animal procedures were approved by Ethical Committee for the Use of Laboratory Animals of Institute for Biological Research “Sinisa Stankovic” (Belgrade, Serbia), as being in compliance with EEC Directive (86/609/EEC) on the protection of animals used for experimental and other scientific purposes. Animals were housed (3–5/cage) in a pathogen-free and climate-controlled environment in polystyrene cages containing wood shavings with free access to standard rodent chow and water with regulated 12-hour light/dark cycles. During period of paralysis, animals were watered manually.

Animals were randomly divided into two groups (control, n = 10 and EAE group, n = 30). EAE was induced with the spinal cord homogenate (prepared from freshly dissected spinal cords) and mixed with an equal volume of complete Freund’s adjuvant containing 0.5 mg/ml Mycobacterium tuberculosis (CFA; Sigma, St. Louis, MO, USA) in a 1:1 ratio. Female Dark Agouti rats (150–200 g) were inoculated with a 100 μl intradermal injection of the prepared emulsion. Immunization was performed under ether anesthesia. The group of age-matched intact animals was used as a control.

The rats were regularly examined, weighed and scored for neurological signs of EAE for 25 days post immunization (dpi), by two independent observers according to standard 0–5 EAE grading scale: 0, unaffected; 1, tail atony; 2, hind limb weakness; 3, complete hind limb paralysis; 4, tetraplegic; 5, moribund state or death.

### Tissue preparation

Rats were deeply anesthetized with Zoletil®50 (Virbac, France; 30 mg/kg *i.p*.). Animals from EAE group were sacrificed at the onset (9 dpi, D9), peak (13 dpi, D13) and end (22 dpi, D22) of disease. Age-matched naïve animals were used as control. Lumbar regions of the spinal cords were used for all further tissue processing.

### Real-time PCR

Lumbar spinal cord segments were collected at D9, D13 and D22 (3/group). In order to minimize degradation of spinal cord samples, anesthetized rats were perfused intracardially with 50 ml of cold (4 °C) saline. Afterwards, the rats were placed on ice, and a laminectomy of the entire lumbar spinal cord was performed. The excised segments were placed in precooled (4 °C) sterile RNase/DNase-free microcentrifuge tubes and immediately flash frozen in liquid nitrogen and kept at −80 °C until RNA extraction. Total RNA was isolated using Trizol Reagent (Invitrogen Life Technologies, Carlsbad, CA, USA). The RNA concentration was assessed by determining OD260, whereas the purity was evaluated based on OD260/OD280 ratio (1.8–2.1, for all samples) and gel inspection. RNA was reverse transcribed using High-capacity cDNA RT-kit (Applied Biosystems, Carlsbad, CA, USA). TaqMan PCR reactions were performed with Assay on- Demand Gene Expression Products (Applied Biosystems, Carlsbad, CA, USA) for ApoE (Assay ID (assay ID Rn00593680_m1) and HMGCR (Assay ID assay ID Rn00565598_m1), and assay-by design gene expression product for CYP46A1, with the following sequences: forward primer (50–30) CAGCT TCCTTCTGGGACATCTC; reverse primer (50–30) GAGCACACGGCCACAAG and FAM-MGB probe (50–30) TTCGTCCTTTTTCCAAAAGT. Reactions were performed in a 25-μl reaction mixture containing 1 × TaqMan Universal Master Mix (Applied Biosystems, Carlsbad, CA, USA) and the cDNA template (10 ng of RNA converted to cDNA). PCR reactions were carried out in the ABI Prism 7000 Sequence Detection System (Applied Biosystems) at 50 °C for 2 min, at 95 °C for 10 min, followed by 40 cycles at 95 °C for 15 s, and at 60 °C for 1 min. The experimental threshold was calculated from the mean baseline fluorescence signal from cycles 3 to 15, plus 10 standard deviations. Each sample was run in triplicate and a mean value of each Ct triplicate was used for further calculation. An endogenous control was included in every analysis to correct the differences in inter-assay amplification efficiency, and the expression of each gene was normalized to actin expression. The obtained results were analyzed by RQ Study Add ON software for 7000 v 1.1 SDS instrument (ABI Prism Sequence Detection System) with a confidence level of 95% (P < 0.05).

### Sudan black

In order to analyze demyelination process during disease development, frozen sections were kept at room temperature, rinsed with propylene glycol, and stained with 0.7% Sudan black B (Sigma-Aldrich, St. Louis, MO, USA) diluted in propylene glycol, for 10 min. The specimens were rinsed with distilled water and mounted in Mowiol (Calbiochem, Millipore, Germany). Normal myelin appeared dark blue to black, while degraded myelin was light blue.

### Oil red O staining

To stain lipids in frozen sections, they were washed in distilled water, rinsed in 60% propylene glycol with subsequent incubation in Oil red O for 15 minutes. Afterwards, the sections were washed in 60% propylene glycol, rinsed with distilled water and mounted in Mowiol (Calbiochem, Millipore, Germany).

### Immunostaining

For immunohistochemical and immunofluorescence analyses spinal cords (4/group) were rapidly dissected on ice and fixed in 4% paraformaldehyde in 0.1 M phosphate buffer (PBS), pH 7.4 for 12 hours at 4 °C. To cryoprotect lumbar regions of spinal cord tissue were immersed into the graded sucrose solutions (10–30% in 0.1 M PBS, pH 7.4). Afterwards, the spinal cords were embedded in OCT in 2-methyl butane (tissue freezing medium) for cryosectioning and stored at −80 °C. Serially cut, coronal sections (20 µm thick) were collected, mounted onto superfrost glass slides, allow drying for 2 h at room temperature and stored at −20 °C until staining. 20 µm thick transverse sections of the lumbar spinal cord (L1-L5) were cut and 6 successive parts of the tissue were placed on the same glass slide. Normal donkey serum (10% solution in PBS; Santa Cruz Biotechnology, Santa Cruz, CA, USA) was used to block unspecific labeling. Single peroxidase immunohistochemistry was obtained using rabbit anti-Cyp46A1 antibody T623 (1:70, a generous gift from Dr. David Russell, University of Texas Southwestern Medical Center, Dallas, USA). Using donkey anti rabbit IgG (Santa Cruz Biotechnology, Santa Cruz, CA, USA 1:200), the immunoreaction products were visualized with 3′3-diaminobenzidine (DAB, Dako, Glostrup, Denmark) according to manufacturer instructions. For double immunofluorescence labeling, sections were incubated with rabbit anti-CYP46A1 antibody T623 (1:70), followed by incubation with the antibodies against specific cell markers: mouse anti-GFAP, as a marker of astrocytes (1:500, clone: 73–240, NIH NeuroMab, Davis, CA, USA), mouse anti-ED1, as a marker of macrophages/microglia (1:200, Abcam, MA, USA), mouse anti-neurofilament 200, as a neuronal marker (1:200, Sigma-Aldrich, St. Louis, MO, USA), mouse anti-Neu N, as a marker of neuronal cell bodies (1:200, Millipore, Billerica, MA, USA) or goat-anti Iba1, as a marker of resident microglia (1:200, Abcam, MA, USA) antibodies. Immune complexes were visualized with donkey anti-rabbit Alexa Fluor 555 (1:250), donkey anti-mouse IgG Alexa Fluor 488 (1:250) and donkey anti-goat Alexa Fluor 488 (1:250) all purchased from Invitrogen (Carlsbad, CA, USA). Nuclei were visualized with Hoechst nuclear staining. The controls for validation of immunohistochemical findings were performed followed by recommendation of Burry^57^ and Hewitt *et al*.^58^ (Supplementary Fig. 1). The sections were mounted in Mowiol (Calbiochem, Millipore, Germany) and captured on Zeiss Axiovert fluorescent microscope equipped with camera and EC Plan-Apochromat, using the Apotome system for obtaining optical sections.

### Western blot

Animals were sacrificed under deep anesthesia; lumbar parts of spinal cords were dissected and pooled (3 animals/group/sample; 3 samples/group). Tissue was homogenized in the isolation buffer (0.32 mol/L sucrose, 5 mmol/L Tris pH 7.4) and centrifuged at 1000 × *g* for 10 min at 4 °C. Resulting supernatant was centrifuged at 12 000 × *g* for additional 30 min and the pellet was resuspended and homogenized in 5 mmol/L Tris, pH 7.4 and kept on −70 °C until use. The protein contents were determined using Micro BCA Protein Assay Kit (Thermo Fisher Scientific, Rockford, USA), according to manufacturer’s instruction.

The samples were diluted 1:1 in 2 × sample buffer (BioRad, USA) and equivalent amounts (20 μg of proteins) were resolved on 10% SDS-PAGE gels and transferred to a PVDF membrane (Roche Diagnostics, Mannheim, Germany). After blocking with 5% non-fat dry milk (BioRad, USA) in Tris buffered saline with 0.05% Tween 20 (TBST) (150 mM NaCl, 50 mM Tris, pH 7.4, and 0.05% Tween 20) for 1 hour at room temperature (RT), the blots were probed overnight at 4 °C with the following antibodies CYP46A1 T623 (1:1,000);ApoE (1:5,000; Calbiochem, Germany), HMGCR (1:300, Millipore, Billerica, MA, USA), all produced in rabbit. The antibodies were diluted in TBST, and milk was added to a final concentration of 3% only for the anti-HMGCR antibody. Following several rinses in TBST, the membranes were incubated for 1 h at RT with the appropriate horse radish peroxidase (HRP)-conjugated secondary antibodies (bovine anti-rabbit, 1:2,000; bovine anti-goat, 1:5,000; both from Santa Cruz, sc-2370 and sc-2350, respectively) diluted in TBST. HRP-immunoreactive bands were visualized by enhanced chemiluminescence (ECL, GE Healthcare) and film (Kodak Biomax) exposure. Each blot was reprobed with rabbit anti-actin antibody (1:10,000; Sigma) diluted in TBST. Signals were quantified densitometrically using Image Quant software (v. 5.2, GE Healthcare) and expressed as relative values (i.e., normalized to the corresponding β-actin signals). Changes in the levels of analyzed proteins were expressed as ratios (fold changes) relative to the appropriate control group.

### Data analysis

All values were expressed as the mean ± SEM for Figure 1, and the mean±S.D. for Figure 2. Differences between the experimental groups were tested using ANOVA followed by Bonferroni test. Statistical significance was set at P < 0.05.

## Electronic supplementary material

Supplementary Information
