# Supplementary material for: Expression profiles of cholesterol metabolism-related genes are altered during development of experimental autoimmune encephalomyelitis in the rat spinal cord
Source: Sci Rep. 2017 Jun 2;7:2702. doi: 10.1038/s41598-017-02638-8 (PMC5457442; doi:10.1038/s41598-017-02638-8)
Supplement: Supplementary file 1 — Supplementary Information [file 41598_2017_2638_MOESM1_ESM.pdf]

Expression profiles of cholesterol metabolism-related genes are altered during development of experimental autoimmune encephalomyelitis in the rat spinal cord

Irena Lavrnja [1], Kosara Smiljanic [1], Danijela Savic [1], Aleksandra Mladenovic-Djordjevic [1], Katarina Tesovic [1], Selma Kanazir [1*] and Sanja Pekovic [1*]

[1] Department of Neurobiology, Institute for Biological Research “Sinisa Stankovic” University of Belgrade, Belgrade, Serbia

SUPPLEMENTARY INFORMATION

Figure S1

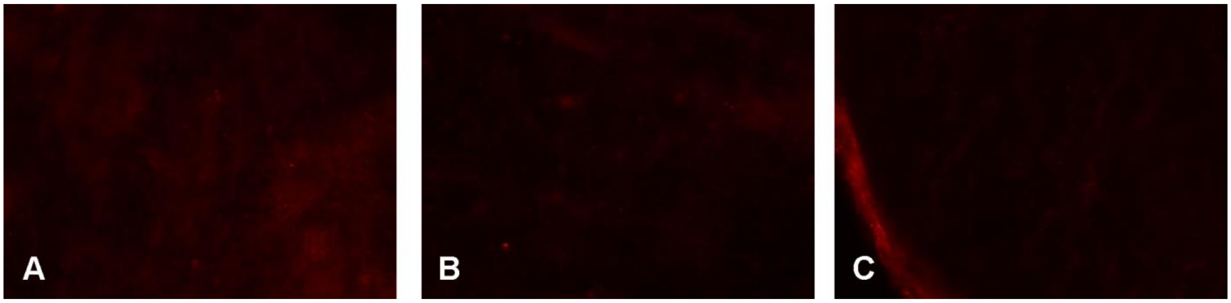

Supplementary Figure 1. Negative control sections. Rabbit serum staining in spinal cords of animals at the onset (A), peak (B) and end (C) of disease.

Figure S2

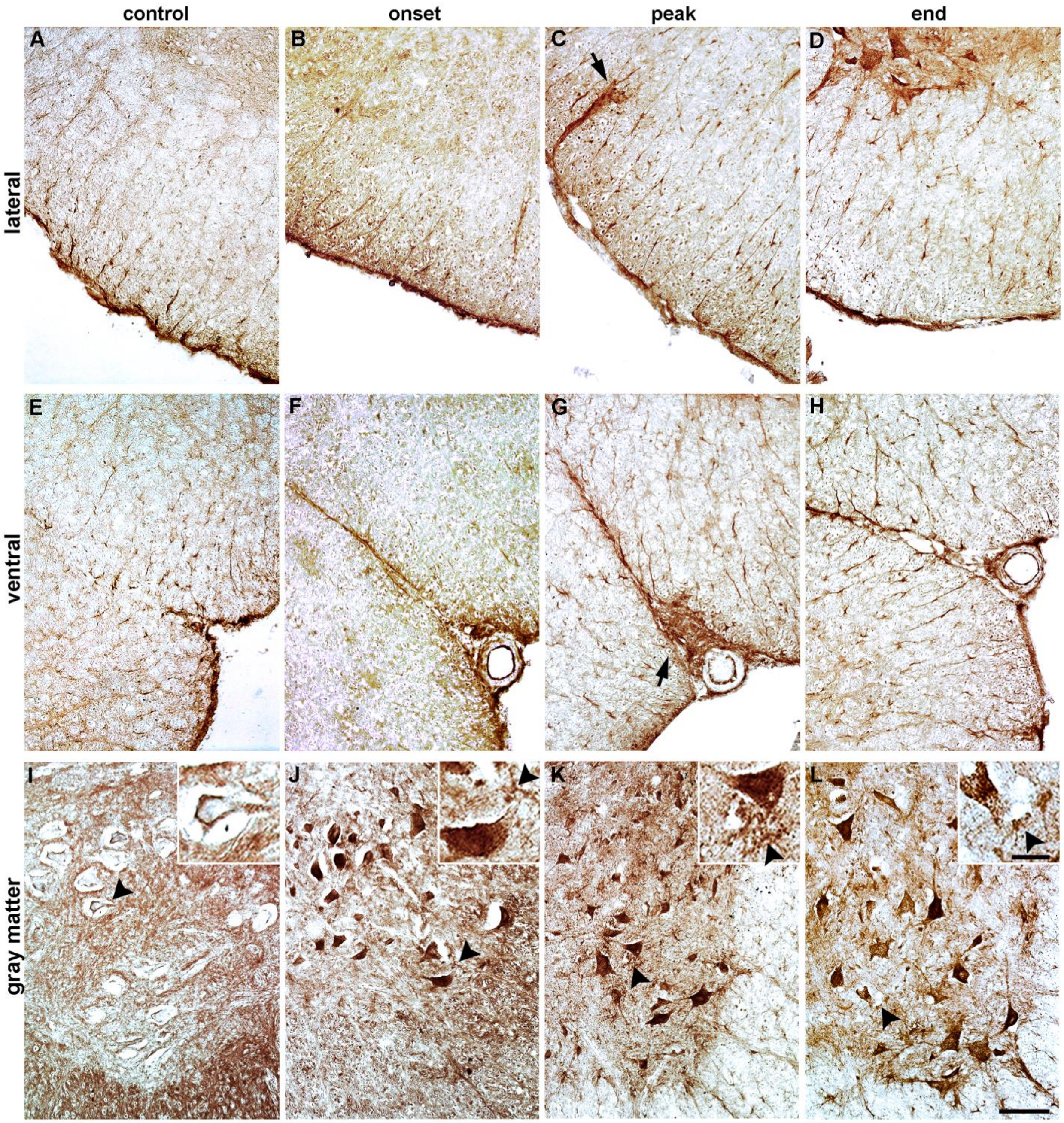

control
onset
peak
end
lateral
ventral
gray matter
A
B
C
D
E
F
G
H
I
J
K
L

Supplementary Figure 2. Cellular localization of the Cyp46A1 expression in different regions of spinal cord is related to disease manifestation. Immunostaining of CYP46A1 in the lateral and ventral parts of the white matter and in the gray matter of rat spinal cord was performed using rabbit anti-Cyp46A1 antibody T623 and was visualized by DAB-HRP staining. (**A, E**) In the control sections and (**B**, **F**) at the onset of EAE a paucity of glia-like CYP46A1^{+} cells were scattered throughout the white matter of lateral and ventral regions of spinal cord. (**C, G**) Intensity of CYP46A1 immunoreactivity in this type of cells was increased at the peak and (**D**, **H**) to a lesser extent at the end of disease both in lateral and ventral regions, respectively. (**I – L,** insets) Analysis of CYP46A1 immunoexpression in the gray matter revealed its co-localization predominantly with neuronal cell bodies. However, (**K,** arrow head, insets) at the peak and (**L,** arrow head, insets) at the end of disease CYP46A1^{+} reactive astrocytes-like cells were noticed. Scale bar = 100 μm for the large panels and 25 μm for the magnified insets.

Figure 3S

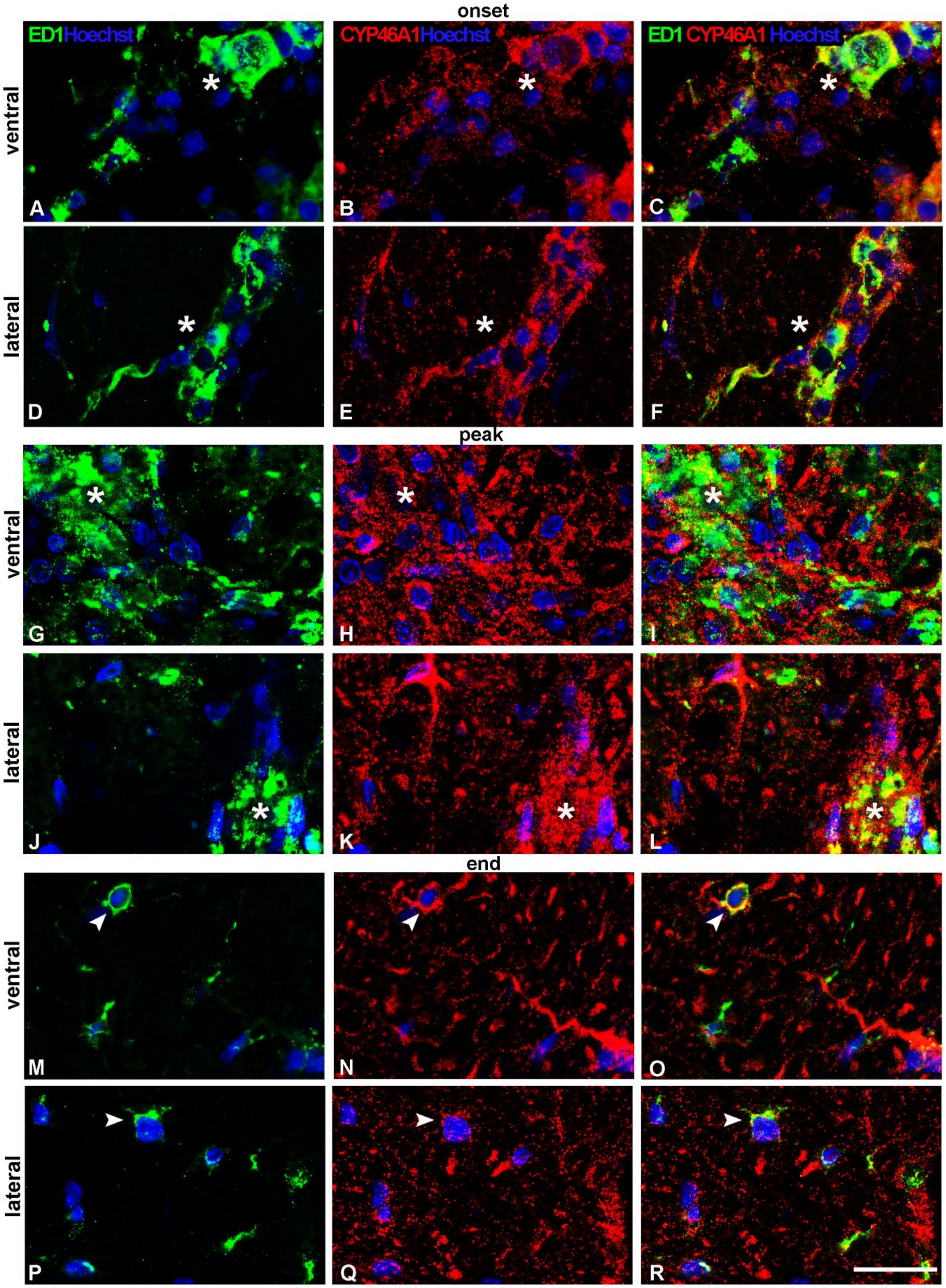
onset
ED1 Hoechst
CYP46A1 Hoechst
ED1 CYP46A1 Hoechst
ventral
lateral
A
B
C
D
E
F
peak
ventral
lateral
G
H
I
J
K
L
end
ventral
lateral
M
N
O
P
Q
R

Supplementary Figure 3. Infiltrating macrophages express CYP46A1 during development of EAE. Co-localization of Cyp46A1 expression (red) with ED1-positive (green) reactive macrophages/ microglia in the ventral and lateral regions of the spinal cords. Hoechst (blue) was used for nuclear staining. (**A-C** and **D-F,** asterisk) At the onset of disease CYP46A1 expression in $ED1^+$ macrophages was detected only in the areas of infiltration. (**G-I** and **J-L,** asterisk) At the peak of disease intensive CYP46A1/ED1 immunoreactivity overlaps with areas of macrophage infiltration and demyelination. (**M-O** and **P-R,** arrow head) At the end of disease, faintly stained $ED1^+$/$CYP46A1^+$ cells were dispersed throughout the white matter. Scale bar = 20 µm.

Figure S4

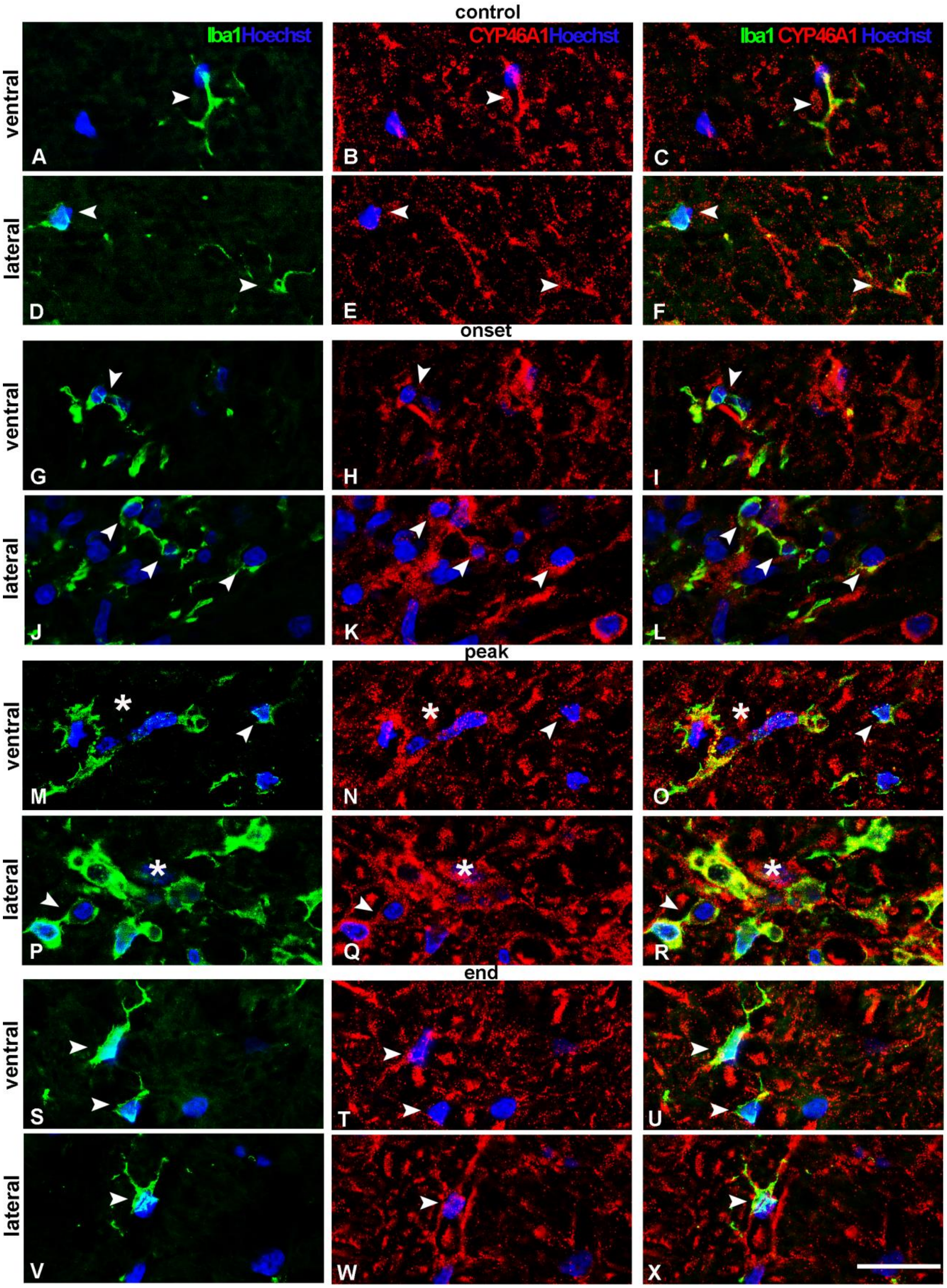
control
Iba1 Hoechst
CYP46A1 Hoechst
Iba1 CYP46A1 Hoechst
ventral
lateral
A
B
C
D
E
F
onset
G
H
I
J
K
L
peak
M
N
O
P
Q
R
end
S
T
U
V
W
X

Supplementary Figure 4. Co-localization of CYP46A1 with Iba1-positive resident microglia correlates with clinical signs of EAE. Double immunofluorescence was used to reveal the presence of CYP46A1 (red) in the Iba1-positive resident microglia (green) in the ventral and lateral funiculus of the spinal cord. Cell nuclei were visualized with Hoechst (blue) staining. (**A-C,** arrow head) In the control sections, ramified $Iba1^+/CYP46A1^+$ microglial cells were scattered through ventral and (**D-F,** arrow head) lateral regions of lumbosacral spinal cord. (**G-I,** arrow head) At the onset of disease, faint CYP46A1 immunoreactivity was infrequently detected in microglial cells in ventral and (**J-L,** arrow heads) lateral spinal cord regions. (**M-O** and **P-R,** asterisk) At the peak of disease strong CYP46A1 immunoreactivity completely co-localizes with a huge number of hypertrophied $Iba1^+$ cells accumulated within the areas of demyelination in the ventral and lateral regions of spinal cord, respectively. (**S-U** and **V-X,** arrow heads) $Iba1^+$ cells obtained resting morphology at the end of disease, and only a few of them express CYP46A1. Scale bar = 20 µm.

Figure S5

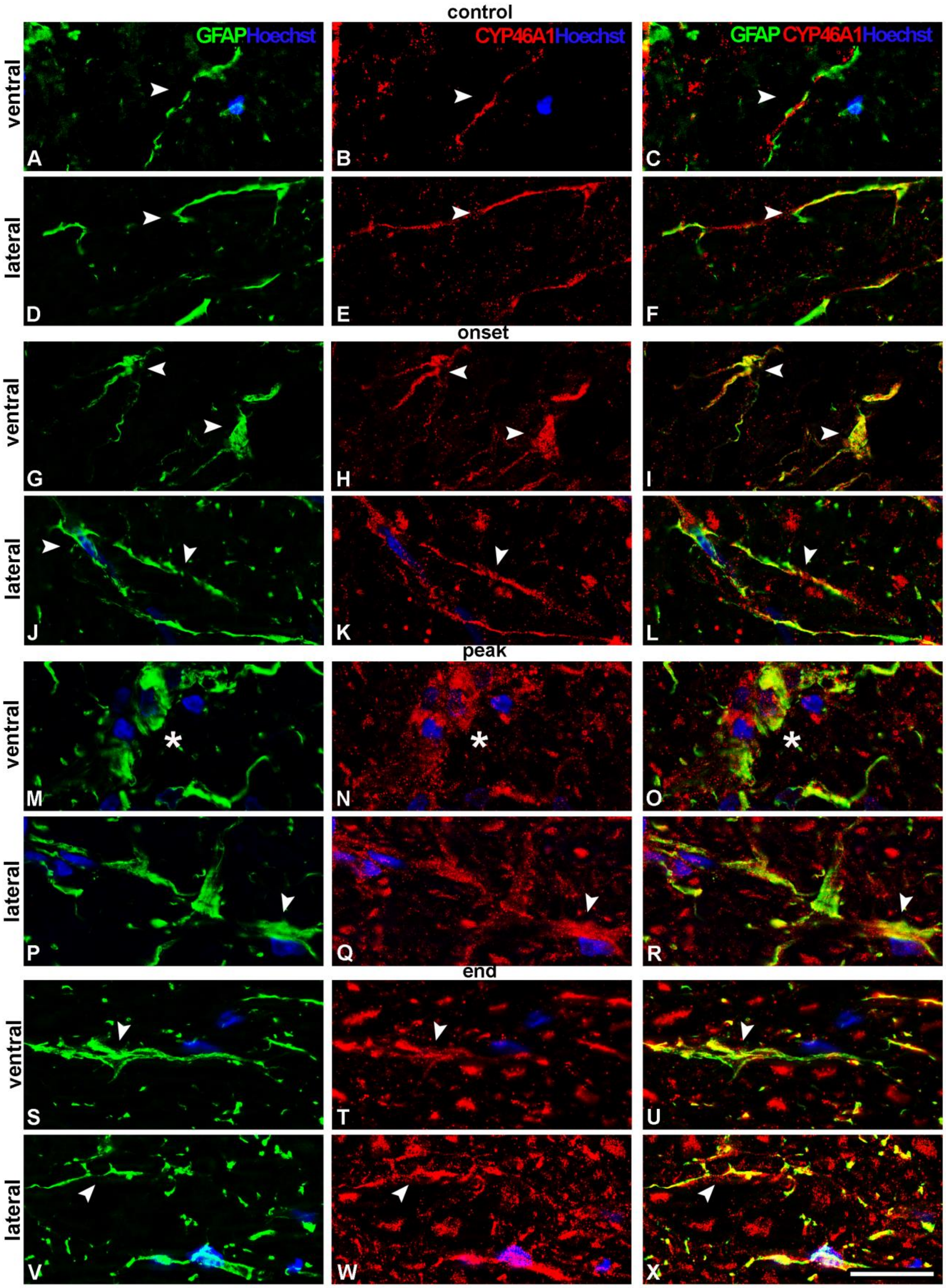
control
GFAP Hoechst
CYP46A1 Hoechst
GFAP CYP46A1 Hoechst
ventral
lateral
onset
ventral
lateral
peak
ventral
lateral
end
ventral
lateral
A
B
C
D
E
F
G
H
I
J
K
L
M
N
O
P
Q
R
S
T
U
V
W
X

Supplementary Figure 5. CYP46A1 co-localize with reactive astrocytes during the course of EAE within the white matter of the lumbar spinal cord. GFAP-positive astrocytes (green) express Cyp46A1 (red) in the ventral and lateral funiculus of spinal cord. Nuclei were visualized with Hoechst (blue) staining. (**A-C**, arrow head) In the control sections of ventral, as well as in the lateral (**D-F**, arrow head) regions of lumbar spinal cord, fibrous astrocytes with the long, thin extensions display CYP46A1 immunoreactivity. At the onset of disease, CYP46A1 immunoreactivity overlaps with (**G-I**, arrow head) GFAP-positive protoplasmic astrocytes within the ventral funiculus and (**J-L**, arrow head) GFAP-positive astrocytes with enlarged cell bodies and long extensions in the lateral regions of the spinal cord. (**M-O**) At the peak of EAE densely packed $GFAP^+$ astrocytes expressing CYP46A1 form the glial scar surrounding the areas of demyelination (asterisk) in the ventral funiculus. (**P-R,** arrow head) $CYP46A1^+$/$GFAP^+$ hypertrophied astrocytes with short processes comprised the lateral regions of the spinal cord. (**S-X,** arrow head) At the end of EAE, hypertrophied astrocytes with elongated processes display overlapping signal with CYP46A1 in both ventral and lateral funiculus. Scale bar = 20 µm.
